# Where to Retire? Experiences of Older African Immigrants in the United States

**DOI:** 10.3390/ijerph19031040

**Published:** 2022-01-18

**Authors:** Manka Nkimbeng, Alvine Akumbom, Marianne Granbom, Sarah L. Szanton, Tetyana P. Shippee, Roland J. Thorpe, Joseph E. Gaugler

**Affiliations:** 1 Division of Health Policy and Management, University of Minnesota School of Public Health, 420 Delaware St SE., Minneapolis, MN 55455, USA; tshippee@umn.edu (T.P.S.); gaug0015@umn.edu (J.E.G.); 2 Johns Hopkins School of Nursing, 525 N. Wolfe Street, Baltimore, MD 21205, USA; aakumbo1@jhu.edu (A.A.); sarah.szanton@jhu.edu (S.L.S.); 3 Department of Health Sciences, Lund University, 22100 Lund, Sweden; marianne.granbom@med.lu.se; 4 Hopkins Center for Health Disparities Solutions, Johns Hopkins Bloomberg School of Public Health, 624 Broadway, Baltimore, MD 21205, USA; rthorpe@jhu.edu

**Keywords:** older adults, African immigrants, retirement, aging-in-place, age-friendliness

## Abstract

Doubling in size since the 1970s, the aging needs of the African immigrant population are not fully understood. This qualitative study examined experiences of aging and retirement planning for African immigrant older adults in the United States (U.S.). Specifically, it explored the factors, processes, and ultimate decision of where these older adults planned to retire. Secondary analysis of semi-structured interviews with 15 older African immigrants in the Baltimore–Washington Metropolitan area was conducted. Data was analyzed using thematic analyses in NVivo. The majority of participants were women, with a mean age of 64. Three overarching themes with ten sub-themes were identified. The themes included: (1) cultural identity, which indicated the participant’s comfort with the U.S. society and culture; (2) decision making, meaning factors that impact participants’ choice of retirement location; and (3) decision made, meaning the final choice of where participants would like to retire. Age-friendliness for immigrant older adults in the U.S. is complex and it includes traditional domains such as physical and sociocultural environment (e.g., housing, transportation, and income). However, immigrant age-friendliness also needs to include wider contextual aspects such as political climate of their country of origin, immigrant status, family responsibilities, and acculturation in the U.S. More research is needed to better understand and facilitate age-friendly environments and transnational aging of immigrant older adults.

## 1. Introduction

Older immigrants make up 13.9% of older adults in the United States (U.S.) and that number is growing [1]. One in five Americans will be 65 or over by 2030 [2]. The aging of current immigrants and the inflow of new older adults are both trends that contribute to the growth of the U.S. older adult population [3,4,5]. The average age of an immigrant in the U.S. increased from 39 years to 45 years between 2000 and 2017 [3]. Most older immigrants are from Latin American countries (38%), followed by those from Asian (31%) and European (24%) countries [1]. Although Africans only make up 6% of the older adult immigrant population in the U.S., almost one fifth of them are over 55 years [6]. There are currently over 2 million African immigrants in the U.S. [6,7].

Migration histories can influence retirement planning and health trajectories. Immigrants migrate for multiple reasons including employment, education, family reunification, or forced migration as refugees or asylum seekers [8,9]. Africans emigrate for three main reasons: better economic opportunities, forced migration, and family reunification [10,11,12]. Most immigrants arrive in the U.S. without health challenges, but the migration and aging process combined with socioeconomic inequity can influence poor health outcomes in older age. Several studies have shown that immigrants are healthier than their U.S.-born peers of the same age [13,14,15,16]. This phenomenon, known as the ‘healthy immigrant effect’ or ‘Hispanic paradox’, is often explained by healthier lifestyles in immigrants’ countries of origin, healthy self-selection at migration, and extensive screening of immigrants before and after arrival into the U.S. [17,18].

Another explanation for why current immigrants might be healthier than their U.S.-born peers is the suggestion that some immigrants may choose to return to their country of origin once their health begins to fail [19,20,21]. There is a growing body of research that suggests that an immigrant’s health worsens with greater length of residence in the U.S. [22,23,24,25,26,27]. Studies of the ‘healthy immigrant effect’ and ‘salmon bias’ amongst Africans are limited, but we reported that foreign-born Black (African and Afro Caribbean immigrants) older adults have better physical function compared to their U.S.-born counterparts [28]. However, aging and retirement plans for older African immigrants are unknown; thus, it is unclear how the current health profile and migration trajectories affect these plans.

In addition to the pursuit of education and economic opportunity, the recent migration of older adults as part of family reunification and refugee admissions has resulted in higher numbers of older adult immigrants [1,29]. These immigration factors can influence long-term health outcomes and well-being, both positively and negatively. For example, refugees or asylum seekers and those who experience pre-migration trauma (being beaten, raped, or seeing other people get hurt or killed) have more depressive symptoms [30,31,32], while those who migrated to join other family members are less likely to experience depressive symptoms [33]. Additionally, older immigrants joining family members may enjoy more social support since many older immigrants reside in extended-family households, especially those who immigrated after the age of 60 [29]. Ethnic enclaves, defined as a geographical area where an ethnic group is spatially clustered and socially and economically distinct from the majority group [34], reportedly have complex effects on the mental health of immigrants [35,36].

Post-immigration trajectories and access to social services may also affect retirement plans. Older immigrants face many linguistic and cultural challenges related to accessing care and support services in the U.S. [29,37,38]. In addition, older immigrants are more likely to have limited incomes, low savings or retirement income, and are ineligible for many federal social service programs including health insurance [29,39,40,41]. In addition to these concerns, loneliness, social isolation, and insufficient use of social services have also been reported by Chinese older adult immigrants [42]. These concerns can be further compounded in undocumented immigrant older adults struggling to avoid deportation [43].

Following immigration, African immigrants are subjected to numerous socioeconomic, cultural, and political challenges that affect their trajectory and wellbeing, and subsequently, retirement plans. The purpose of this study was to describe the experiences of aging and retirement planning for African immigrant older adults in the U.S. Specifically, it explored the factors, processes, and ultimate decision of where the older adults plan to retire. Understanding this can facilitate health care and retirement planning not only for Africans but also for other immigrant groups who might share similar reasons for migration and health trajectories.

Theoretical Model: This study is guided by the socio-ecological model [44,45,46]. This model postulates that health and health behaviors are influenced by multi-level factors. These factors include the individual (biological and personal history), relationships (closest social circle that includes partners and family members), community (settings such as schools and neighborhoods), and societal (broad societal factors such as cultural norms) [45] factors. The final level of the original model includes internal and external elements of time and historical elements that has been updated to include policies [44,47]. These factors interact across these different levels to influence specific health behaviors [46]. This model is used to explore and describe the factors that influence older African immigrants’ desires, plans, and decision-making surrounding aging in the U.S. and retirement planning.

## 2. Materials and Methods

### 2.1. Study Design

This is a secondary qualitative analysis of data collected as part of a larger mixed-methods study of functional limitations [27]. Inclusion criteria were: (1) ≥50 years, (2) born in Africa, (3) ability to communicate in English, (4) lived in the U.S for ≥6 months, (5) Black race, (6) cognitively intact as determined from the form—the six-item screener [48], and (7) have at least one mobility limitation based on National Health Interview Survey Mobility questions [49]. The study protocol was approved by an academic medical institution ethical review board (IRB) and the study was conducted from 2018 to 2019. As a convergent mixed methods study [50], quantitative and qualitative data collection phases occurred concurrently. Note that the current study focuses on data from the qualitative strand.

### 2.2. Recruitment and Data Collection

Detailed recruitment and data collection procedures have been reported elsewhere [27,51]. Briefly, study participants were recruited from community-based organizations (CBOs), word of mouth, and social media. Data collection occurred at community-based organizations and events following the organization’s leadership approval and subsequent IRB approval of that recruitment site. Some participants completed mailed surveys and home data collection was also conducted as needed. Trained research assistants obtained oral informed consent from participants using an IRB approved consent script and then administered the study survey.

Interview participants were purposively selected from the quantitative sample using participants’ acculturation and discrimination scores [51]. Acculturation was measured with the modified Psychological Acculturation Scale (PAS) [52] and discrimination measured with the Everyday Discrimination Scale (EDS) [53,54]. The PAS measures changes that may occur during the acculturation process with questions such as “which group(s) of people do you feel the most comfortable?” The PAS is divided into two dimensions; one for U.S., and the other for country of origin. Scores ranged from 0–4 with high scores indicating greater affiliation to that country dimension. The EDS assesses experiences of discrimination during everyday activities. Scores ranged from 0–27 with higher scores indicating more experiences of discrimination. To obtain a diverse sample, interview participants were selected based on whether they had high or low scores on the acculturation country domains, and whether they were below or above the current sample median discrimination scores.

Twenty-six participants were identified for the qualitative interviews. Interviews were scheduled progressively, and 15 participants were interviewed. One-on-one interviews were conducted in participants’ homes by the first author, a female African immigrant doctoral candidate at the time. Only the participant and interviewer were present during the interviews. A semi-structured interview guide was developed specifically for the study which included questions such as “Do you plan to spend the rest of your life in the United States? Why?” “Do you plan to go back to (use country of origin) or a different country? Why” (see Appendix A). Interview times ranged from 46–120 min and the average time to complete an interview was 78 min. Preliminary analyses during data collection informed saturation (when no additional themes were derived) and recruitment termination.

### 2.3. Data Analysis

Audio recordings were transcribed by a professional transcription service, and data analyzed using thematic analysis [55]. The first and second authors analyzed the data. In phase 1, analyses began with each coder reading through the same four randomly selected transcripts for familiarization and understanding of the context. In phase 2, two interviews were selected for initial coding by both coders using an inductive approach. They identified a list of codes including for example “immigrants are never fully American”, “why people want to stay”, “time to go”, “desire to split time”, “physical strength to work”, “differences in healthcare”. In Phase 3 and 4, the first and second author collated the codes and searched for preliminary themes, which were then reviewed together during data analyses meetings. The emerging themes and sub-themes were developed through an iterative process. For example, two overarching themes were identified preliminarily (“staying versus going back” and “planning for retirement”) but, following further discussion, the coding team subsequently agreed that cultural identity was a unique theme that informs but is different from the decision making and decision made final themes. In Phase 5, the identified themes and sub-themes were named and defined. The codes within each emerging theme were then listed into a codebook that was used to code all 15 interviews in NVivo 12. No additional codes were identified at this stage. In Phase 6, identified themes and analysis was finalized and results reporting began. Finally, a deductive approach was used to identify the level within the socio-ecological model where each theme or sub-theme fits.

### 2.4. Trustworthiness and Rigor

Notes were taken during the analyses to document insights arising from the data. Confirmability was achieved by having each interview coded by two independent coders and meetings of the coding team to discuss emerging themes, define, and name the overarching themes obtained. Consensus on final themes was achieved across all coders. Reflexivity and credibility were achieved through memoing during individual coding and discussion of these notes in analysis meetings. The core analytic team comprised of two Black African immigrant (foreign-born) researchers (first and second authors), but peer debriefing happened in consultation with a White Swedish qualitative research expert (third author). Findings are reported in accordance with the Consolidated Criteria for Reporting Qualitative Studies guidelines [56].

## 3. Results

Table 1 presents sociodemographic characteristics of each participant by pseudo name, age, country of origin, length of residence in the U.S, acculturation and discrimination scores. Of the 15 participants, the mean age (±SD) of the sample was 63.9 (±5.4) years. Nine participants were female. Eight participants were married, and nine had a bachelor’s degree or higher. Ten participants had health insurance coverage, four participants were from households with incomes between $0–39,999, four were from households with $40,000–$80,000, two lived in households with incomes over $80,000, and three did not know their household incomes or preferred not to answer. Nine participants were currently employed in some capacity. Participants had lived in the U.S. for from less than a year to 37 years. The mean African culture subscale score was 4.5 (range 0–5), that of the U.S. culture subscale was 3.4 (range 0–5), and the average discrimination score was 9.3 (range 0–27).

Three themes—cultural identity, decision making, and decision made—and ten sub-themes were identified from these data. Pseudo-names were used to protect the identity of study participants.

### 3.1. Cultural Identity

Participants described their degree of comfort and fit with U.S. society and discussed how they identified themselves. These choices and descriptions represented a complex process that included their perceived acceptance and knowledge of U.S. culture and individual behavior. Regardless of their identity, participants expressed reasons for why they identified as they did and their desire for respect was an important factor.

Not-American: Among these older adults, seven individuals identified themselves as Not-American or by their ‘country of origin’ primarily. For some, it was a conscious choice not to let their identity be influenced by the new country, while for others it was out of necessity. Three important reasons could be observed for why participants described themselves as Not-American or African, as well as why they reported not fitting in the U.S. culture. These reasons included: (1) not understanding the culture; (2) not feeling accepted by the U.S. society and culture, especially in relation to their accents; and (3) actively choosing not to change who they were to fit in. For example, Laura, who had lived in the U.S. for over 28 years stated, *“I cannot beAmerican because they don’t accept you. They will never accept people, and I cannot change my accent because I love the way I speak”* while Gloria who had lived in the U.S. for over 37 years, stated “*…African as a whole. I think I would fit there … I don’t know that much about American [sic]. Their society is different from our own*”.

However, some older adults described themselves as Not-American because they had not lived in the country long enough to know the culture yet. Joshua, who had been in the U.S. for less than 1 year, stated, “*To me, for now, I’m a Cameroonian. And if I’m integrated in the American society, that is when I can think that…and so, yes, I cannot now say I’m American*”. Additionally, participants described identity preferences in relation to the activities they chose to engage in and noted that they would strive to fit-in in professional or work events and activities.

Dual-Identity: Six participants’ descriptions reflected a dual identity as ‘African-American’ or ‘country of origin—American’. These participants who identified themselves as both African and American noted that they were Africans first; as Philip stated, “*I see myself as a Ghanaian-American, because, one, I have to accept that I’m a Ghanaian. And then, two, I have to accept that I’m on the soil of America*”. In keeping with their American identity, participants described friendships, profession and work, and other activities they engaged in within the U.S. society and culture (paying bills, working, and voting). Additionally, participants actively maintained their African cultural practices, including cooking traditional dishes, wearing traditional clothing, and attending cultural events such as meetings and country-affiliated churches. Some participants described it as an active process of maintaining a dual identity, stating that it was conscious choice, while for others it appeared to be temporary because they currently lived in the U.S.

American identity: This identity was not common. One participant, Rose, described herself in a much broader context than just American, noting, “*I would not fit in both. I always think international. I will never stay in a place that’s all-black or all-white. I like a mixture*”. Another participant, Magdalene, stated, *“I have accepted being an American, so for me, now, it’s trying to live comfortably here. The only way you can do that is if you become an American. That’s the only part that bothers me that you have to give up your identity to be able to live in the world*”. This choice, which appeared to be worrisome to Magdalene, also appeared to be temporary to enable her to live comfortably in her new country. Although she explicitly described herself as American, she also engaged in African cultural practices including eating African foods, speaking her tribal language, and wearing traditional African clothing. Finally, having U.S. citizenship status also influenced some participants to identify as American.

### 3.2. Decision Making

This theme entails how participants dealt with a complex decision-making process, including why they chose their particular place(s) for retirement. They discussed several factors that interacted with their chosen cultural identity and fit with the U.S. to influence the choice of where they will retire. Although we describe these factors individually, the decision-making process involved a complex interplay amongst these factors that subsequently influenced participants’ final decisions.

Social and community engagement: Participants discussed how the level of engagement they had with their community influenced their feeling of belonging or ‘fitting- in’ in the U.S. system and their retirement decision. Participants who said they would be staying typically described the importance of continuously engaging in the community. However, participants also described how community engagement activities could pose challenges to comfortably retiring in the U.S. compared to their country of origin where they had a higher social status or more independence. For example, Cletus stated, “*You join certain groups, you have to make some contributions. You have to socialize. You have to maybe have meetings where you drive. You see, I don’t have a car. Maybe if I didn’t do medicine, I could have easily fit in this society*”.

Additionally, the possibility to fit in within a desired community and social group in retirement was also a consideration, especially for those who planned to retire in their country of origin. As described by Peter: “*One of the reasons why ’I’ve decided to do a PhD program is this: I come from a group which almost all of them are professionals and have PhDs and other higher education levels. I will be a misfit with them if I go back with a Masters program*”. The ability and plan to further his education to fit within his chosen social circle in retirement was an important consideration in his decision to retire in Ghana.

Family ties and responsibility: Love for family and caring responsibilities here or back in the country of origin was the most prevalent factor described that influenced retirement plans. Laura, who had decided to stay in the U.S., noted, “*The thing is I have the children here. They are going to school. And I have the job to do. So when am I going home? This is home*”. Other participants, such as Philip, who had made the decision to move back, explained, “*My wife wants to go home…*” describing that a partner’s preference was vital to one’s own decision making. Additionally, some participants discussed how their property and homes also influenced the decision to stay in the U.S. or return to their country of origin.

Current health and wellbeing: These older adults described how current health status, their perception of age, functional ability, and well-being had influenced the decision on where to retire. Veronica, who was dependent on her son for help, noted, “*… As I told you, I’ve used my life until now. I’m burnt out*” when she was determining to stay in the U.S., indicating that her current health played a role in the decision to stay. Others noted that they would like to go back when they were still healthy to be able to engage in the community through politics, while Gloria (whose health was already failing) also identified how poor health of family members in Liberia (lack of possibilities to get help and care when needed) had influenced her decision to stay in the U.S., “*My younger sister, she is sick, she is almost paralyzed, she can’t take care of me. Then why I am going there for? I can’t go there*”.

Health care access: The perception of easy access to health care facilities and services was also described as a factor in the decision for where participants chose to retire. They reported that health care and providers in the U.S. were much better than in some of their African countries of origin. Many participants who decided to return to their country of origin also discussed that they wanted to return to the U.S. in the future when in need of health care. Magdalene, a 65-year-old from Liberia, expressed her fear: “*But right now, home is not conducive to me. My girlfriend went last year, after 32 years too, and she died four days after she got there… So all of us are afraid to go home right now, because they have no medical facilities*”.

Political climate: Some participants described how war or political crises in their countries of origin, which once influenced immigration to the U.S., was currently affecting visits to their country of origin as well as their retirement plans. They expressed fear of getting arrested or general fear for safety because of these crises. Joshua from Cameroon stated frankly: “*I prefer to stay here. My life has no duplicate; that is, I go there and it goes off*”. Furthermore, they discussed how their immigration status, such as asylum status, had resulted in an inability to travel back to country of origin now or later in life.

### 3.3. Decision Made

This theme showed that the participants had already decided where they wanted to spend the rest of their lives. They identified two options: staying in the U.S. or returning to their country of origin. About half of the participants had decided to stay in America, and the other half planned to return to their country of origin. No matter what option they had decided, participants were planning on frequently visiting their country of origin or the U.S.

Staying in America: Older adults who decided to stay had come to the conclusion that this was home for the foreseeable future. Many factors such as supporting their children, feeling safe in the U.S., an established U.S. identity, or assimilation appeared to influence this decision. One participant, Laura, reported, “*This is home. I’m an American citizen. It took many years to do that*”. Another participant noted that an important factor was the love they felt for the U.S., specifically, the county where she lived. Participants who planned to stay in the U.S. seemed certain of this decision, but many, such as Rose, described that they plan to visit family in their country of origin. However, two people in this group described scenarios which indicated that there could be a change in their decision to stay if the political situation in their country of origin or their immigration status would change.

Returning to country of origin: Many participants had decided to return to Africa. They had begun planning what they would do to earn income, contribute to society, remain busy after retirement, or a combination of these. Additionally, participants already had a timeline or anticipated time for when their retirement would begin, and some had begun building (or were preparing to build) homes to live in, in their country of origin. Peter, 62 years old, from Ghana declared: “*I will go to Ghana, or have two homes. Definitely! I will go and then I will come back [to the U.S]*”. Just like their counterparts who planned to retire in the U.S., these participants seemed certain of their future plans to move back to their country of origin, except for one participant who described a scenario where his decision might change to include coming back to visit the U.S. after retirement if his immigration status were to change.

**Figure 1:**
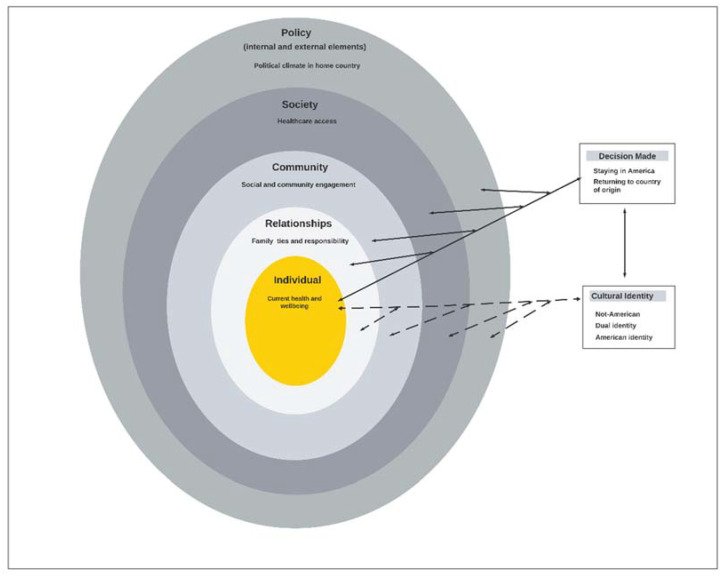
Older African immigrant decision making about where to retire.

Figure 1 describes how these themes interact and influence factors across different levels within the socio-ecological model. Cultural identities interact with individual, relationships, community, and societal factors and subsequently influenced the decision about where the older adult plans to retire. For example, individual factors such as greater length of residence affect identity as an American, which subsequently affects the decision to retire in the U.S. Alternatively, family cohesion, spousal preferences, and relationships with kids and grandkids influenced a decision to retire in the U.S., whereas attachment to family members in country of origin influences the decision to retire in Africa. Political climate determined by policies in country of origin also influence decision making around retirement for these older adults. As depicted in Figure 1, these participants described factors across all levels of the socio-ecological model that interacted in complex ways to influence retirement planning and decision making.

## 4. Discussion

The purpose of this study was to explore older African immigrants’ experiences of aging in the U.S. and understand retirement decision making for a rapidly growing population. In addition to self-reported cultural identity and the perception of ‘fitting-in’ in the U.S. society and context, five factors were identified to influence the choice of retiring here or returning to country of origin. Regardless of where they planned to retire, older adults described their plans to go back and forth to the U.S. (for those retiring in country of origin) or to their country of origin (for those retiring in the U.S.). Retirement planning is an important aspect of successful aging and age-friendly environments and should be tailored to the needs of its residents.

Older African immigrants’ decisions or plans for retirement revealed that participating older adults were current or future transnational immigrants, frequently traveling between their African country of origin and the U.S. [57]. Transnationalism is the process where immigrants build and maintain social fields to link themselves with their home country, and it involves many levels of transactions [58]. Though limited, older studies show that many African immigrants in the U.S and Canada plan to retire in Africa. Owusu (2003) reported that 91% of Ghanaians in Canada had intentions to return to their country of origin, while Agyemang and Takyi (2001) found that 63% of African immigrants in the U.S. had intentions to return to their country of origin [59,60]. Moreover, our findings deepen the knowledge about the reasoning behind those decisions. In preparation for retirement and returning to Africa, African immigrants had begun investments and building houses (homes) in their countries of origin [59,60,61]. Therefore, older African immigrants appeared to have been preparing for a long period of time and expressed a dual loyalty; loyalty to both their country of origin and current country of residence.

The descriptions of older African immigrants’ health and aging plans are more complex than suggested by the ‘healthy immigrant effect’ and ‘salmon bias’. Although limited evidence suggests that older Black immigrants (including Africans) have better function and cardiovascular disease risk factors compared to U.S.-born Blacks [28,62], there is also evidence to the contrary. O’Connor and colleagues (2014) reported that African immigrants had poorer cardiometabolic health compared to African Americans [63]. More importantly, growing evidence suggests that any potential or perceived immigrant health advantage is lost in older age with longer residence in the U.S. [23,24]. In this sample, older African immigrants’ descriptions revealed that health and well-being played a more complex role in their decision of where to retire. Some older adults chose to return to their country of origin following retirement when they were older and could no longer work, while others chose to remain in the U.S. because their health had already failed and they feared that family members in Africa would not be able to care for them. Although African immigrants may have better health at younger ages when they arrive in the U.S, their health likely worsens with time in the country, which influences their retirement plans.

The reasoning surrounding retirement planning has not been explored in African immigrants. Our findings suggest that African immigrants’ perceptions about healthcare infrastructure and systems in each country played an important role. In a World Health Organization study of the community perceptions and perspectives of health systems in Africa with over 10,000 participants from 10 countries, over two-thirds of the sample reported that health service delivery was inadequate [64]. The primary reasons for this inadequacy were the unavailability of drugs and equipment, while financial and transportation barriers were also identified as factors that limited access to healthcare [64]. Participants in our study described the inability of family members to care for them, while others reported plans to come back to the U.S. for health care. This likely reflects an understanding of the limited access and inadequacies of the current health care systems in many African countries.

Accessibility to health care and caregiving is described as a factor in the decision-making process for these older adults with chronic diseases and disability who may need frequent medical care, which might not be easily accessible in their country of origin. Taking into consideration access to healthcare, social support and housing options are known to be important factors to older adults when planning for retirement and reasoning related to aging-in-place or relocation [65,66]. Our findings with immigrant older adults adds the complexity of having to consider these options in two countries (current country of residence and African country of origin). Moreover, deciding where to grow older and whether the current home will be suitable for aging-in-place is known to be a long process that is filled with ambivalence [66,67,68,69]. In our findings, participants do not seem to share the same ambivalence. In addition to its effects on the process of decision making, perhaps being an immigrant also plays a role in the timing and speed of decision making for where the immigrant older adult will retire.

Current work on facilitating age-friendly homes, cities, and environments has been carried out in developed countries, while little or no work has been done in developing countries, many of which are African countries. Age-friendly homes or communities are places that foster active and healthy aging by ensuring that they can maintain the current intrinsic capacity and functional abilities of the individuals residing in these settings [70]. The sociocultural context in many African countries can support some domains of age-friendliness, while at the same time present challenges for others. Core indicators of age-friendliness include the accessible physical environment and inclusive social environment [71]. Domains in these core indicators that are described by these older adults included engagement in social-cultural activity (such as food, clothing, and events), participation in family decision making, and access to housing, including the ability to build homes in their country of origin. Additionally, it is generally known that Africans have a positive attitude toward older people, including a culture for respecting older adults that can facilitate age-friendlessness. However, the limited access to healthcare in country of origin, transportation, and financial barriers described above will present challenges to these older adults who have resided in the U.S. for long and have become used to a certain level of health care access and delivery.

Creating age-friendly environments is challenging for older adults; creating them for older adults who plan to live simultaneously in two continents presents even more challenges. Our findings indicate that age-friendliness for immigrant older adults is complex and exceeds the traditional domains such as physical and sociocultural environment, and includes international aspects and factors such as political climate in their country of origin, immigrant status, family responsibility, and healthcare infrastructure in country of origin. There are currently no African countries in the Global Network for Age-friendly Cities and communities [70]. In addition to facilitating stronger health care systems in African countries, governments have to facilitate an increase in accessibility of public health transportation, public spaces and buildings, paid employment, and economic security that can improve age-friendliness and facilitate successful return and residence of aging African immigrants from the U.S. and other Western countries.

This study has some limitations. Firstly, this study was a secondary data analysis of a larger study with a different overarching purpose. Secondly, while we attempted to recruit participants from all African countries, study participants were primarily from five African countries (Cameroon, Ghana, Nigeria, Liberia, and Sierra Leone). Participants in this study could communicate in English, and these results might not reflect the experiences of non-English speaking older African immigrants and those from countries outside of the five above. Additionally, while this manuscript describes factors that can influence desires, plans, and decision-making surrounding aging in the U.S. and retirement, this is likely not an exhaustive list. Notwithstanding these limitations, this in-depth qualitative exploration of the U.S. aging experience of African immigrant older adults offers insights into the retirement plans of this growing population that can inform tailored health education and planning, both for individuals and health systems.

Although some studies have suggested that immigrants are healthier than their U.S.-born peers and that sick immigrants will choose to return to their countries of origin, this study showed that the health and retirement decision making for older African immigrants are more intricate and aging-in-place in one age-friendly environment is not the preferred option. Participants had different conceptualizations of their identities and many factors influenced their choice of where to retire. Subsequently, these older adults were (or planned to become) transnational immigrants choosing to travel frequently and visit their African country of origin or the U. S. after retirement. Being transnational immigrants adds another layer of complexity to the process of facilitating an age-friendly environment for older African immigrants.

These findings also add to the growing literature on transnational aging and can inform future directions for research. Transnational aging is the process of organizing and coping with life that is not limited to the context of a single country. As observed in the current study, immigrants consider multiple factors including health and different cultural context in retirement decision making [72]. Yet more research is needed to explore how social factors and constrains, space, time, knowledge, and the portability of retirement benefits influence these decisions [71,72,73]. Additionally, an understanding of how the advancement of media, technology, and transportation affects these plans [74], especially in the context of other factors that can impact travel, such as the global COVID-19 pandemic, is needed. As a nascent field, more research is needed to understand how macro-level (politics), meso-level (organizations) and the micro-level (family/individual) factors interact to influence transnational aging [75].

## Figures and Tables

**Table 1:** Characteristics of the sample (N = 15).

Pseudo Name	Sex	Age	Length of Stay	Marital Status	Country of Origin	Immigration Status	Level of Education	Employment Status	Household Income	Health Ins.	Acculturation (U.S Score)	Acculturation (CoO Score)	Discrimination Score
Judith	Female	64	30	Widowed	Cameroon	Asylum seeker	Bachelor’s	Part-time	$20,000–$39,999	No	2.5	3	5
Paul	Male	60	4	Married	Cameroon	Asylum seeker	HS/ GED	Part-time	Under $20,000	No	3	3	8
Janet	Female	58	6	Married	Cameroon	Permanent resident	Bachelor’s	Full-time	$20,000–$39,999	Yes	2.8	3.3	16
John	Male	61	22	Married	Cameroon	Citizen	Master’s	Full-time	$80,000–$99,999	Yes	1.3	3.5	11
Joshua	Male	68	0	Married	Cameroon	Asylum seeker	<HS	Retired	Don’t Know	No	2.1	2.7	7
Cletus	Male	74	20	Married	Cameroon	Permanent resident	Master’s	Retired	$40,0000–$59,999	Yes	0.7	4	14
Veronica	Female	68	28	Widowed	Cameroon	Citizen	Bachelor’s	Retired	Under $20,000	Yes	2.3	3	4
Mary	Female	59	22	Widowed	Ghana	Citizen	Associates	Full-time	$40,0000–$59,999	Yes	1.5	3	10
Philip	Male	62	14	Married	Ghana	Visa holder	HS/GED	Seeking	Prefer not answer	No	3.3	3.8	3
Peter	Male	62	17	Married	Ghana	Citizen	Master’s	Full-time	$60,000–$79,000	Yes	3	3	12
Gloria	Female	72	37	Widowed	Liberia	Citizen	No formal ed.	Retired	Don’t Know	Yes	2	4	3
Magdalene	Female	65	33	Separated-Divorced	Liberia	Permanent resident	Master’s	Retired	$40,0000–$59,999	Yes	4	4	22
Felicia	Female	65	3	Married	Nigeria	Permanent resident	Bachelor’s	Part-time	Under $20,000	Yes	1.7	3.8	7
Rose	Female	61	13	Separated-Divorced	Sierra Leone	Citizen	Masters	Full-time	$40,0000–$59,999	Yes	3	3.17	3
Laura	Female	52	28	Widowed	Sierra Leone	Citizen	Master’s	Part-time	$80,000–$99,999	No	2.5	4	23

CoO = Country of origin.

## Appendix A. Older African Immigrant Health Study Interview Guide

Phase 1
101. Every immigrant has a story of how they ended up in the United States. What is your story? a. *Probe: What country are you from?* b. *Probe: How long have you lived here?* c. *Probe: Did you come directly from the country where you were born? If not please explain.*
102. How was your life in your country of origin? *(please use participant’s country from above)* a. *Probe: What work did your do?* b. *Probe: How was your health?* c. *Probe: Did you live in the city?*
103. Tell me about your decision to come to the America? a. *Probe: When did you decide to come to the U.S.?* b. *Probe: Why did you decide to move to the U.S.?*
104. How is your life now? a. *Probe: What work do your do?* b. *Probe: How is your health?* c. *Probe: Do you live in a community with many people from your country of origin? (please use participant’s country from above)*
Phase 2
201. I would like to know more about different aspects of your health. We will start with how you care for yourself. I’d like you to walk me through your day from the moment you wake up and when you go to sleep for the night. Let’s start with when you wake up…”. a. *Probe: Describe any difficulties you have with your morning care- bathing/taking a shower, using the toilet, brushing your teeth.* b. *Probe: Describe any difficulties you have with preparing and eating meals (bringing the groceries in, standing to cook, opening cans, chopping spices, eating etc.* c. *Probe: Describe any difficulties you have doing chores and cleaning the house (dishes, sweeping/vacuuming, and yard work.)*
202. Some people have medical problems such as arthritis or are taking medications that make it hard for them to do things around the house. Please describe if you think your health makes it difficult for your do things around the house and the community? a. *Probe: How does your medical problems affect how you to do things in the house or get around outside the house?* b. *Probe: How do your medications affect your ability to do things around the house?* c. *Probe: Describe if you have any injuries that make it difficult to do you things around the house? Tell me how you sustained the (name patient identified injury)?* d. *Probe: Describe if this is different from when you were in (name country of origin)*
203. Some people use things like a cane or reacher to help them walk or get things done around in the house. Please tell me if there are things or people that help you take care of yourself or the house? *(Get list of people and items)* a. *Probe: Describe how (pick item or person) helps. (Ask this for all people/items on the list).* b. *Probe: Describe if this is different from when you were in (name country of origin)*
204. Can you tell me how you go to places such as the doctor’s office, church or market? a. *Probe: How did you get here (use name of current interview site).* b. *Probe: Describe if you have difficulty getting in a car or on the bus?* c. *Probe: Please tell me if this is different from how you were in (use country of origin from above)*
Phase 3
301. As an immigrant, so many things about my life have changed since I came here. And you, how has your life changed since you moved to the U.S? a. *Probe: Please describe if there are things you are doing differently now, why? (E.g. health care, eating habits, exercise, social relationships and the way your visit your friends and family, language etc.*
302. Please describe any ways you think that your life has gotten better since you came to the United States? a. *Probe: Have you achieved the goal (reason) that brought you to the United the States? How did you do this?*
303. Please describe anyways you think your life has gotten worse since you came to the United States? a. *Probe: Is your life better off because you moved to the United States?*
304. How often do you go back to your country of origin? *(please use participant’s country from above)* a. *Probe: Do you go back to your (country of origin) as often as you would like?* b. *Probe: Do you bring back things from your home country when you visit? If yes, identify these things?*
305. Compare your health now to how it was in country of origin *(use name of participant’s country of origin).* a. *Probe: Please describe any changes in your medical problems (including medications)* b. *Probe: Please describe any changes to the way you see doctors or go to the hospital?*
Phase 4
401. In every country, one can find discrimination (unfair treatment) against people based on their religion, race, ethnicity or tribe, gender and occupation. What forms of discrimination happen in *(use country of origin from above)*? a. *Probe: Did you experience any discrimination there? What form did it take?* b. *Probe: How did that experience make you feel?*
402. Please tell me if you have experienced any discrimination in this country? a. *Probe: Please describe the types of discrimination you have experienced in this country?* b. *Probe: Please tell me how this (these) experiences made you feel.*
Phase 5
501. Please describe if you think your health is worse or better now than it was in *(use country of origin)*
502. How do you think you fit in in the American society? a. *Probe: Please discuss if you feel more American or African (name country of origin) and why.*
503. Thinking about your future, tell me if you have plans to move again? a. *Do you plan to spend the rest of your life in the United States? Why?* b. *Do you plan to go back to (use country of origin) or a different country? why*
504. Is there anything else about you, how you care for yourself and home in this country that you would like us to know?
Follow up questions to the Interviewer
601. How do you think the interview went? a. *Probe: Describe if participant appeared comfortable with questions.* b. *Probe: Did participant have enough time to answer all questions?*
602. How do you relate to participants experiences? a. *Probe: Describe any experiences you share with the participant.* b. *Probe: Is participant from the same country as you?* c. *Probe: Describe any experiences you have that are different from participant*
